# Nomograms to predict 2-year overall survival and advanced schistosomiasis-specific survival after discharge: a competing risk analysis

**DOI:** 10.1186/s12967-020-02353-5

**Published:** 2020-05-06

**Authors:** Guo Li, Lifei Lian, Shanshan Huang, Jinfeng Miao, Huan Cao, Chengchao Zuo, Xiaoyan Liu, Zhou Zhu

**Affiliations:** Department of Neurology, Tongji Hospital, Tongji Medical College, Huazhong University of Science and Technology, 1095 Jiefang Avenue, Wuhan, Hubei 430030 China

**Keywords:** Advanced schistosomiasis, Nomogram, Advanced schistosomiasis-specific survival, Overall survival, Competing risk

## Abstract

**Background:**

The prognosis of patients with advanced schistosomiasis is poor. Pre-existing prognosis studies did not differentiate the causes of the deaths. The objectives were to evaluate the 2-year overall survival (OS) and advanced schistosomiasis-specific survival (ASS) in patients with advanced schistosomiasis after discharge through competing risk analysis and to build predictive nomograms.

**Methods:**

Data was extracted from a previously constructed database from Hubei province. Patients were enrolled from September 2014 to January 2015, with follow up to January 2017. OS and ASS were primary outcome measures. Nomograms for estimating 2-year OS and ASS rates after discharge were established based on univariate and multivariate Cox regression model and Fine and Gray’s model. Their predictive performances were evaluated using C-index and validated in both internal and external validation cohorts.

**Results:**

The training cohort included 1487 patients with advanced schistosomiasis. Two-year mortality rate of the training cohort was 8.27% (123/1487). Competing events accounted for 26.83% (33/123). Older age, splemomegaly clinical classification, abnormal serum DBil, AST, ALP and positive HBsAg were significantly associated with 2-year OS. Older age, splemomegaly clinical classification, abnormal serum AST, ALP and positive HBsAg were significantly associated with 2-year ASS. The established nomograms were well calibrated, and had good discriminative ability, with a C-index of 0.813 (95% CI 0.803–0.823) for 2-year OS prediction and 0.834 (95% CI 0.824–0.844) for 2-year ASS prediction. Their predictive performances were well validated in both internal and external validation cohorts.

**Conclusion:**

The effective predictors of 2-year OS and ASS were discovered through competing risk analysis. The nomograms could be used as convenient predictive tools in clinical practice to guide follow-up and aid accurate prognostic assessment.

## Background

Schistosomiasis is a serious parasitic disease caused by blood flukes (trematode worms) of the genus *Schistosoma* [1]. As one of the 17 neglected tropical diseases listed by the World Health Organization, it presents the greatest global burden of disease, leading to 200 million infections and threatening 800 million residents in 78 countries worldwide [2, 3]. Schistosomiasis is caused by *Schistosoma japonicum* in China. Over the past seven decades, Chinese government has made great strides toward reducing the prevalence and incidence of schistosomiasis, largely through a strategy based on chemotherapy and snail control [4]. According to 2017 national report of schistosomiasis, 37601 cases with *S. japonicum* infection and 29407 patients suffered from advanced schistosomiasis were reported [5]. Advanced schistosomiasis is regarded as the most severe form of schistosomiasis. This debilitating condition is associated with splenomegaly, ascites, portal hypertension, liver fibrosis and cirrhosis and gastro-oesophageal variceal bleeding, leading to disability or even death [6]. The dwarfism and colonic tumoroid proliferation sub-types are rarely found at present while ascites and splenomegaly subtypes are still common in Hubei, a province by the Yangtze River in Central China [7].

Cases with advanced schistosomiasis are registered and managed independently because the disease results in high levels of mortality and disability as well as poor quality of life. The prognosis of patients with advanced schistosomiasis is poor. The death is usually triggered by the upper gastrointestinal tract bleeding, hepatic failure, spontaneous bacterial peritonitis and so on [6, 7].

Our previous study has shown that the 2-year mortality of patients with advanced schistossomiasis was as high as 8% in which the 2-year all-cause death after discharge served as the primary outcome [8]. However, the practice in prevention and cure of advanced schistosomiasis has shown that these patients did not necessarily die as results of advanced schistosomiasis or relevant complications. For example, due to the long disease duration, patients’ condition recurrent attacks and low cure rate, the negative emotions can lead to suicide when they become severe [9, 10]. Furthermore, some patients died by accident due to lack of social support, or from other primary diseases. In our study, non-advanced schistosomiasis-specific deaths (NASDs) and advanced schistosomiasis-specific deaths (ASDs) are regarded as two competing events in case of survival analysis. NASDs, such as primary gastric cancer, pancreatic cancer and suicide, account for a sizeable proportion. The risk of NASDs also increases as age increases. The competing risk analysis must be considered when the absolute percentage of competing events is more than 10% [11]. Failure to take the presence of competing risks into account may result in misleading conclusions in the prognostic prediction of advanced schistosomiasis [12]. Nonetheless, there is no study addressing the incidence of ASD and NASD events in patients with advanced schistosomiasis so far. Different from the all-cause death outcome in our previous study [8], the survival outcome in this study was evaluated by competing risk analysis to take NASDs into account. Competing risk analysis can more adequately capture the real cause-specific survivals of the patients with advanced schistosomiasis after discharge.

The post-discharge follow-up is of considerable value for patients with advanced schistosomiasis to determine whether further treatment be needed [13, 14]. Thus, patients with advanced schistosomiasis should receive periodic follow-up after discharge, including physical and imaging examination. Proper clinical predictive tool on patients’ survival outcome, such as nomogram, could help guide follow-up and aid accurate prognostic assessment. It is well known that personalized prevention and treatment are based on the accurate prognostic evaluation, in which clinical prognostic factors need to be clearly illustrated. For example, the competing risk analysis has been widely used in cancer research and competing risk nomograms have recently been developed for cancers such as lung cancer, thyroid cancer and renal cell carcinoma [15–17]. However, competing risk nomogram for predicting mortality risk of patients with advanced schistosomiasis is still lacking.

To fill this important gap of knowledge, we studied a large population-based cohort of patients with advanced schistosomiasis from Jingzhou to construct the predictive nomograms and externally validated in Huangshi cohort [8]. Increased awareness of the nomograms, accurate prediction of the OS and ASS rates and adequate management of prognostic factors through appropriate interventions could improve the patients’ prognosis [18]. Therefore, competing risk nomograms, which could provide accurate individualized prognosis predictive tools in clinical practice, were utilized based on the revealed prognostic factors. This means that when evaluating the prognosis of these patients after discharge, the competing risks of ASD and NASD have to be weighed against each other based on the effective nomograms [19].

## Methods

### Study population and outcome

This population-based retrospective cohort study used medical information from a previously constructed database of patients with advanced schistosomiasis from Jingzhou and Huangshi cities of Hubei Province, China. The inclusion and exclusion criteria were as the same as our previous study [8]. The training cohort and external validation cohort was also as the same as previous study [8], while the internal validation cohort included 700 patients who were randomly selected from the training cohort. The discharge time window for the patients was from September 2014 to January 2015. All of them were followed up to January 2017. The primary outcome measures were 2-year overall survival (OS) and advanced schistosomiasis-specific survival (ASS) after discharge. OS was restricted to the duration from the date of discharge to death or last follow-up, with no restriction on the cause of death. ASS was also restricted to the duration from the date of discharge to death or last follow-up, with the rate equivalent to 1 minus advanced schistosomiasis-specific mortality.

### Baseline characteristics

16 baseline candidate variables were chosen based on literature review and clinical practice. The chosen variables should be representative, low-cost and easily obtained in clinical settings, thus serving as conveniently translated predictors [20]. The candidate variables included: age, gender, nourishment status and so on [8]. Patients received routine laboratory tests on admission. Serum samples were collected and clotted at room temperature, then centrifuged at 3500 r/min for 10 min to estimate the levels of 8 serum biomarkers. The baseline demographic and clinical information were extracted using standard questionnaires. Clinical classification was identified according to WS261-2006 [6].

### Statistical analysis

The differences of the 16 variables between training and internal validation cohorts were compared. Results were exhibited as mean ± standard deviation (SD) and median (interquartile range, IQR) for continuous variables depending on the normal or non-normal distribution of data, while categorical variables were expressed as numbers (percentages). Student’s t test was used to explore the differences between the normally distributed variables, while the Mann–Whitney U test was used to test the non-normal distributed variables. The categorical variables were compared using the Chi squared test. By restricted cubic splines (RCS) method, we have found the nonlinear association between serum ALB and the survival outcome [8]. The serum ALB was categorized to ≤ 45 g/L and >45 g/L groups based on the RCS curve. TBil, DBil, ALT, AST and ALP were categorized into normal and abnormal groups based on their medical reference value, respectively.

The OS was analyzed using the Kaplan–Meier method. The log-rank test was used to compare the differences between groups. ASD and NASD were regarded as two competing events. The combined effects of the significant variables on OS and ASS were evaluated by proportional hazard analyses of Fine and Gray’s model. Univariate and multivariable Cox proportional hazards regression models were then used for variables selection. The existence of multi-collinearity between co-variates were determined by the VIF values > 5 and tolerances < 0.2, in order to detect the potential interactions between the selected covariates through univariate analysis. Multivariate Cox proportional hazards model was then applied using backwards elimination. The results were presented as hazard ratios (HRs) and 95% confidence intervals (CIs).

Variables with statistically significant differences in the multivariate Cox proportional hazard model (log-rank test, *P* < 0.05) were chosen to build the nomograms. The performance of nomogram was measured by concordance index [C-index, equivalent to area under receiver operating characteristic curve (AUROC)] and assessed by calibration curves in both internal and external validation sets. A higher C-index indicates better ability to separate patients with different survival outcomes. The calibration curves were used to compare the predicted probability with the observed probability. Bootstraps with 500 resamples were applied to reduce the overfitting bias.

Statistical analyses were performed using SPSS version 22.0 software (SPSS Inc., Chicago, IL, USA) and R version 3.5.2 software (The R Foundation for Statistical Computing, Vienna, Austria. http://www.r-project.org). The R packages “survival”, “survminer”, “cmprsk”, “rms”, “Hmisc”, “lattice”, “Formula”, “ggplot2”and “splines” were applied in data analyses.

## Results

### Patient characteristics

The 16 selected variables did not significantly differ between the training and internal validation cohorts (all *P* values >0.05), indicating the reliability of internal validation cohort construction and the comparability of the two cohorts (Table 1). Of the 123 patients who died in 2 years after discharge, 33 cases died from non-advanced schistosomiasis specific causes, such as suicide, car accident and primary gastric cancers. In our study population, in fact, NASD events accounted for 26.83% (33/123). The mean age was 62.89 ± 10.38 years and females accounted for 36.3%. General nourishment status accounted for 70.07% (1042/1487) and 35.84% (533/1487) of the patients underwent splenectomy. Similarly, the other patients’ characteristics of training and internal validation cohorts were also shown in Table 1.

**Table 1:** Clinical and demographic characteristics of the samples

Variables	Training (N = 1487)	Internal validation (N = 700)	Statistics	P value
Demographic characteristics				
Age (years), mean ± SD	62.89 ± 10.38	62.97 ± 9.96	t = −0.164	0.870*
Gender (Males/females)	948/539	464/236	χ2 = 1.335	0.248^#^
Nourishment status (Well/general/poor)	351/1042/94	171/486/40	χ2 = 0.626	0.731^#^
Clinical characteristics				
History of splenectomy (None/yes)	954/533	461/239	χ2 = 0.603	0.437^#^
Comorbidities (None/cardiovascular/digestive/others)	712/167/328/280	360/75/146/119	χ2 = 2.526	0.471^#^
Clinical classification (Splenomegaly/Ascites)	176/1311	97/603	χ2 = 1.780	0.182^#^
Course of disease (years), median (IQR)	5.00 (3.00–8.00)	5.00 (2.25–8.00)	z = −0.375	0.707^$^
Frequencies of ascites (times), median (IQR)	6.00 (4.00–9.00)	6.00 (3.00–9.00)	z = −1.144	0.253^$^
Survival outcome (Death/survival)	123/1364	52/648	χ2 = 0.460	0.498^#^
Laboratory test indices				
TBil (μmol/L), median (IQR)	15.59 (11.43–20.80)	15.5 0 (11.40–21.00)	z = 0.229	0.819$
DBil (μmol/L), median (IQR)	4.70 (3.20–6.87)	4.70 (3.20–6.95)	z = −0.118	0.906^$^
ALT (µ/L), median (IQR)	26.35 (19.00–38.14)	26.00 (18.09–38.99)	z = −0.100	0.920^$^
AST (µ/L), median (IQR)	36.2 0 (28.20–48.00)	36.00 (28.00–48.04)	z = −0.255	0.799^$^
ALP (µ/L), median (IQR)	94.00 (73.00–125.00)	92.00 (73.00–124.00)	z = −0.427	0.669^$^
ALB (g/L), median (IQR)	40.90 (36.00–44.20)	40.78 (36.31–44.10)	z = −0.038	0.969^$^
HBsAg (Negative/positive)	1219/268	578/122	χ2 = 0.115	0.735^#^
AFP (Negative/positive)	1452/35	680/20	χ2 = 0.492	0.483^#^

The data distribution type of TBil, DBil, ALT, AST, ALP and ALB was skewed distribution through normality test

*TBil* total bilirubin, *DBil* direct bilirubin, *ALT* alanine aminotransferase, *AST* aspartate aminotransferase, *ALP* alkaline phosphatase, *ALB* albumin, *HBsAg* hepatitis B surface antigen, *AFP* alpha fetoprotein

*Student’s *t* test; ^#^Chi squared test; ^$^Mann–Whitney U-test

### Univariate, multivariate Cox proportional hazards regression and competing risk analyses

Two-year OS rate of the study cohort was 91.73%. As shown in Table 2, older age (HR = 1.066, 95% CI 1.044–1.089, *P *< 0.001), splemomegaly clinical classification (HR = 1.797, 95% CI 1.127–2.866, *P* = 0.041), abnormal serum DBil (HR = 1.853, 95% CI 1.074–3.198, *P* = 0.027), serum AST (HR = 1.643, 95% CI 1.089–2.477, *P* = 0.018), ALP (HR = 1.675, 95% CI 1.110–2.529, *P* = 0.014) and positive HBsAg (HR = 1.854, 95% CI 1.213–2.834, *P *= 0.004) were significantly associated with 2-year OS through multivariate Cox proportional hazards regression analyses (Table 2).

**Table 2:** Univariate and multivariate analyses of overall survival and advanced schistosomiasis-specific survival

		Overall survival						Advanced schistosomiasis-specific survival					
		Univariate analysis			Multivariate analysis			Univariate analysis			Multivariate analysis		
		HR	95% CI	*P*	HR	95% CI	*P*	HR	95% CI	*P*	HR	95% CI	*P*
Age (years)		1.086	1.042–1.132	0.000	1.066	1.044–1.089	< 0.001	1.062	1.042–1.083	< 0.001	1.091	1.063–1.120	< 0.001
Gender	Males/females	0.881	0.605–1.281	0.507			NI	0.881	0.605–1.281	0.507			NI
Nourishment status	Well/general/poor	1.064	0.754–1.502	0.722			NI	1.064	0.754–1.502	0.722			NI
History of splenectomy	None/yes	0.651	0.437–0.970	0.035			NS	0.651	0.437–0.970	0.035			NS
Other disease	None/cardiovascular/digestive/other	1.033	0.941–1.133	0.492			NI	1.032	0.942–1.133	0.492			NI
Clinical classification	Ascites/splenomegaly	1.778	1.73–2.80	0.013	1.797	1.127–2.866	0.041	1.778	1.13–2.797	0.013	1.904	1.106–3.279	0.020
Course of disease (years)	≤4/>4	0.854	0.599–1.218	0.383			NI	0.854	0.599–1.218	0.117			NI
Frequencies of ascites (times)	<5/≥ 5	0.834	0.581–1.199	0.327			NI	0.834	0.581–1.199	0.327			NI
TBil	Normal/abnormal	3.070	2.153–4.378	<0.001			NS	3.070	2.153–4.378	<0.001			NS
DBil	Normal/abnormal	3.691	2.589–5.261	<0.001	1.853	1.074–3.198	0.027	3.691	2.589–5.261	<0.001			NS
ALT	Normal/abnormal	1.253	0.838–1.875	0.272			NI	1.253	0.838–1.875	0.272			NI
AST	Normal/abnormal	2.468	1.718–3.546	< 0.001	1.643	1.089–2.477	0.018	2.468	1.718–3.546	< 0.001	2.193	1.342–3.683	0.002
ALP	Normal/abnormal	2.763	1.890–4.040	< 0.001	1.675	1.110–2.529	0.014	2.763	1.890–4.040	< 0.001	1.650	1.031–2.640	0.037
ALB	≤45/>45 g/L	0.311	0.158–0.614	0.001			NS	0.311	0.158–0.614	< 0.001			NS
HBsAg	Negative/positive	1.758	1.184–2.610	0.005	1.854	1.213–2.834	0.004	1.758	1.184–2.610	0.005	1.857	1.124-3.068	0.016
AFP	Negative/positive	3.671	1.862–7.236	<0.001			NS	3.671	1.862–7.236	< 0.001			NS

*HR* hazard ratio, *CI* confidence interval, *NS* not significant, *NI* not included

Two-year ASS rate of the study cohort was 93.95%. Older age (HR = 1.091, 95% CI 1.063–1.120, *P *< 0.001), splemomegaly clinical classification (HR = 1.904, 95% CI 1.106–3.279, *P* = 0.020), abnormal serum AST (HR = 2.193, 95% CI 1.342–3.683, *P* = 0.002), ALP (HR = 1.650, 95% CI 1.031–2.640, *P* = 0.037) and positive HBsAg (HR = 1.857, 95% CI 1.124–3.068, *P *= 0.016) were significantly associated with 2-year ASS through multivariate Cox proportional hazards regression analyses (Table 2).

**Fig. 1:**
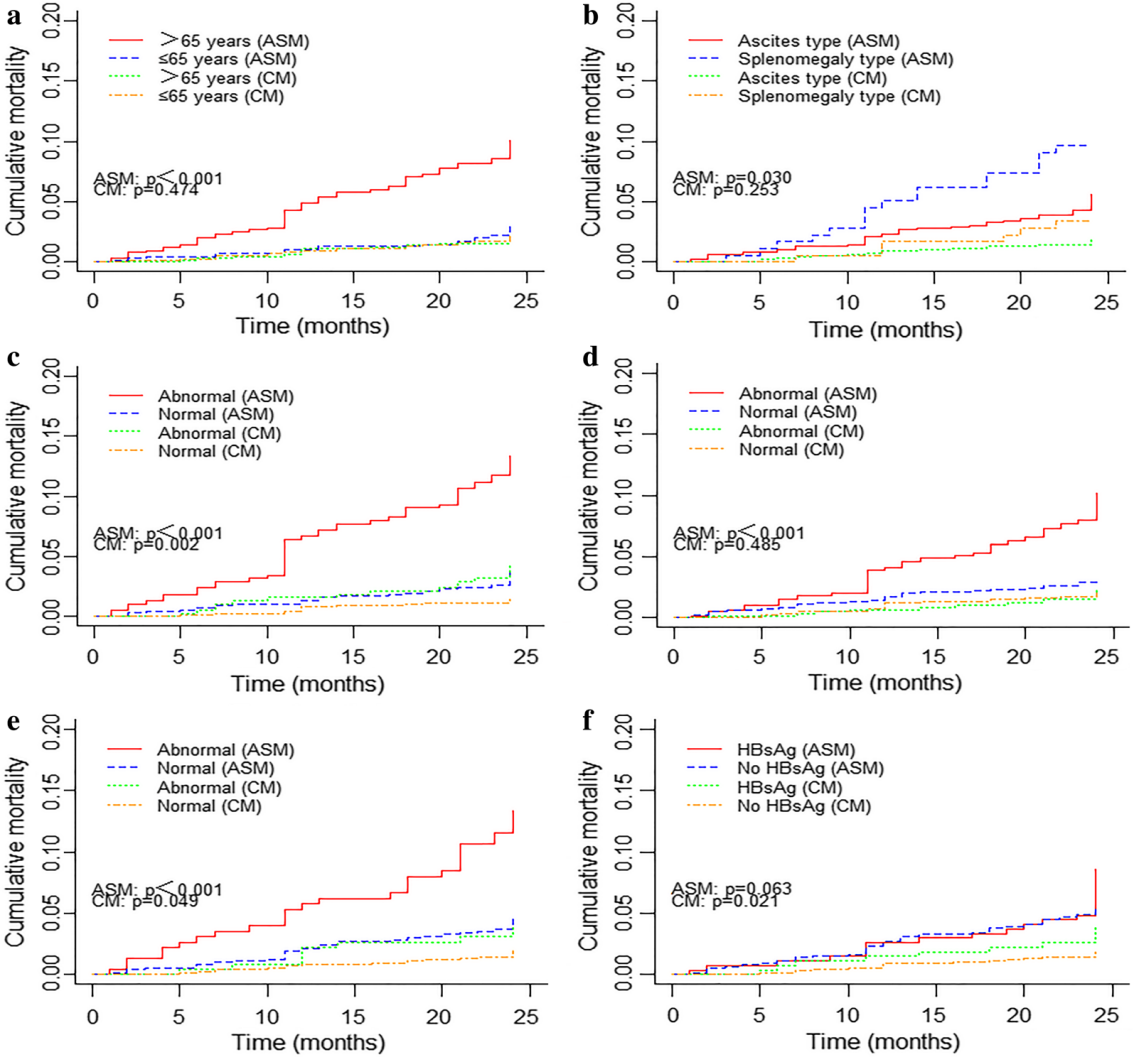
Cumulative incidence estimates of death for patients with advanced schistosomiasis by patient characteristics: **a** Age; **b** Clinical classification; **c** DBil; **d** AST; **e** ALP; **f** HBsAg

**Fig. 2:**
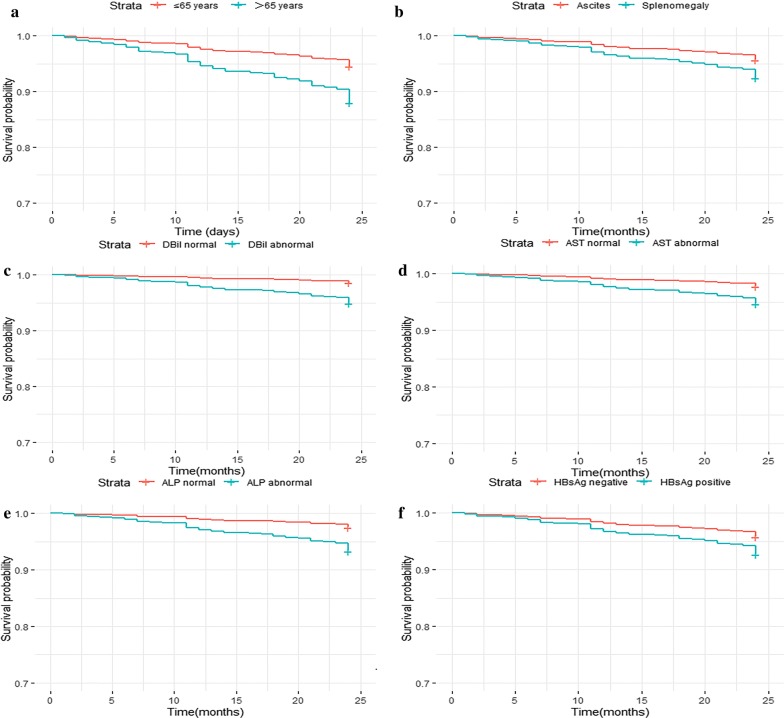
Overall survival rates according to patient characteristics: **a** Age; **b** Clinical classification; **c** DBil; **d** AST; **e** ALP; **f** HBsAg

The adjusted competing risk analysis showed that the cumulative incidences of advanced schistosomiasis specific mortalities (ASM) were significantly higher in patients when they had older ages (*P* < 0.001), splenomegaly type (*P* = 0.03), presence of abnormal DBil (*P *< 0.001), AST (*P *< 0.001) and ALP (*P *< 0.001). The cumulative incidences of competing mortalities (CM) were significantly higher in patients when they had abnormal DBil (*P* = 0.002), ALP (*P *= 0.049) and positive HBsAg (*P *= 0.021) (Fig. 1). Moreover, the Kaplan–Meier curves showed that the overall survival rate were significantly lower in patients who had older ages (*P* < 0.001), splenomegaly type (*P* = 0.01), abnormal DBil (*P* < 0.001), AST (*P* < 0.001), ALP (*P* < 0.001) and positive HBsAg (*P* = 0.005) (Fig. 2).

### Construction and validation of the nomograms for 2-year OS and ASS

**Fig. 3:**
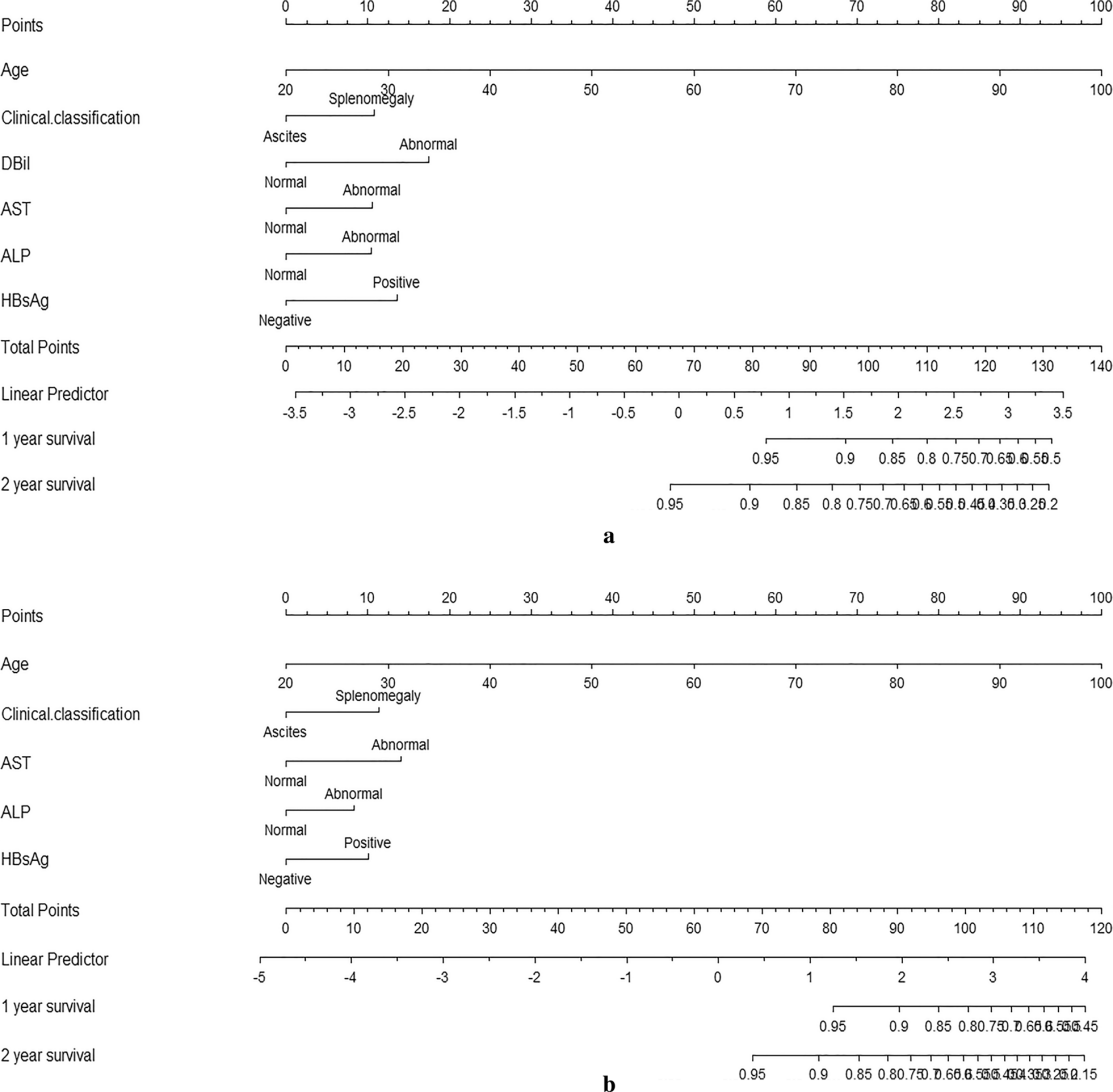
Nomograms predicting 2-year OS (**a**) and ASS (**b**) of patients with advanced schistosomiasis. *OS* overall survival, *ASS* Advanced schistosomiasis specific survival

All the independent predictors of 2-year OS and ASS in the whole study cohort were integrated into the nomograms. Figure 3 illustrates the predictive nomograms for the 2-year OS and ASS rates. A patient’s probability of individual survival can easily be calculated by adding the scores for each selected variable. Each variable is projected upward to the value of the small ruler (Points) to get the score of each parameter. The higher the score is, the worse the prognosis is. The sum of all small rulers is the total score (Total Points).

**Fig. 4:**
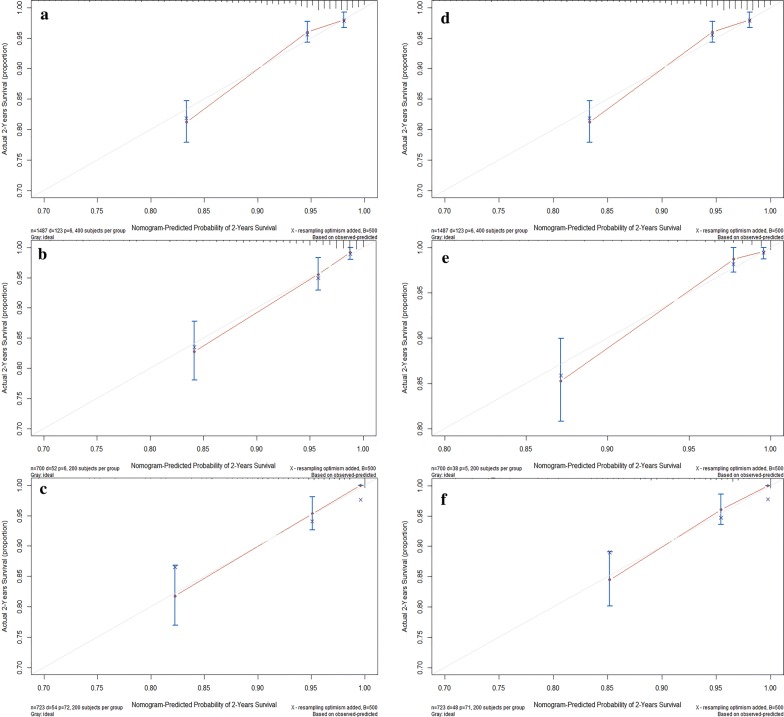
Calibration plots of the nomogram for 2-year OS prediction of the training set (**a**), internal validation set (**b**) and external validation set (**c**), and for 2-year ASS prediction of the training set (**d**), internal validation set (**e**) and external validation set (**f**). X-axis represents the nomogram-predicted probability of survival; Y-axis represents the actual OS probability. A perfectly accurate nomogram prediction model would result in a plot in which the observed and predicted probabilities for given groups fall along the 45-degree line. Dots with bars represent nomogram-predicted probabilities along with 95% confidence interval. *OS* overall survival, *ASS* advanced schistosomiasis-specific survival

The nomograms demonstrated good prdictive performance for OS, with a C-index of 0.784 (95% CI 0.773–0.795) in training set, 0.908 (95% CI 0.890–0.926) in internal validation set and 0.816 (95% CI 0.806–0.826) in external validation set. The nomograms for ASS prediction which we generated based on Fine and Gray’s model also showed good accuracy, with a C-index for the ASS prediction of 0.816 (95% CI 0.805–0.827) in training set, 0.851 (95% CI 0.834–0.868) in the internal validation set and 0.803 (95% CI 0.7894–0.819) in the external validation set. Calibration plots for 2-year OS and ASS rates showed optimal agreements between predictive values calculated by the nomogram and the actual observations in training, internal and external validation sets (Fig. 4). Guided by nomograms, we can accurately predict the prognosis based on the different characteristics of each patient.

## Discussion

The disease burden of schistosomiasis is mainly attributed to the advanced stage, associated with liver fibrosis and cirrhosis, ascites, portal hypertension, enlarged spleen and gastroesophageal varices, leading to disability, loss of workforce and self help ability or even death [21–23]. Previous studies have clearly showed that advanced *schistosomiasis japonica* is associated with high morbidity and mortality, poor self-reported quality of life and heavy disability [7, 8]. However, the special survival analysis literature about advanced *schistosomiasis japonicum* is rare. In this study, we evaluated the 2-year survival outcome for patients with advanced schistosomiasis after discharge using competing risk approach in order to evaluate the prognosis more accurately, in which death from other causes were not censored, but treated as a competing risk failure event. We have presented the cumulative incidence of advanced schistosomiasis specific death. Cumulative incidence function (CIF) is an unbiased estimate for probability of death outcome which reflects the mortality patterns actually observed [16]. Nomograms based on a model that includes patients’ demographic and clinical characteristics could provide an accurate individualized prediction tool which is extremely useful to guide follow-up and aid accurate prognostic assessment. This is the first study to implement competing risk analysis and build nomograms for patients with advanced schistosomiasis based on Fine and Gray’s proportional sub-distribution hazard model. The internal and external validation results have shown a favorable discrimination and calibration because the C-indexes are higher than 0.70 [24]. The nomograms are easy to use in clinical practice because the variables incorporated in the model can be easily obtained from routine clinical work and the principle is readily comprehensible.

The results have shown that older age, splenomegaly clinical classification, abnormal serum DBil, AST, ALP and positive HBsAg were significantly associated with 2-year OS rate after discharge. Meanwhile, older age, abnormal serum AST, ALP and positive HBsAg were significantly associated with 2-year ASS after discharge. Older age, which is associated with an increasing rate of comorbidities, means a significant decline in bodily functions and thus resulting in higher mortality rate [25, 26]. Splenomegaly type patients have many complications after splenectomy, especially combined with hepatic encephalopathy and rebleeding which may also bring about higher mortality risk than ascites type patients [27, 28]. Serum ALB level is influenced by fundamental chronic liver dysfunction and mainly reflects the liver’s protein synthetic capability associated with severity of ascites. There is a high risk of developing further complications of hepatic cirrhosis such as Hepatorenal syndrome (HRS) and spontaneous bacterial peritonitis [29, 30]. Even though the statistic was not significant in multivariate Cox regression analysis (*P* = 0.055), the further study of the ALB role in survival outcome of patients with advanced schistosomiasis was essential. Serum ALP is a hydrolytic enzyme that dephosphorylates and transphosphorylates molecules including nucleotides (adenosine triphosphate, adenosine biphosphate), pathogen-associated molecule patterns and danger-associated molecule patterns [31]. The serum ALP level was reported as a prognostic variable which was integrated into Chinese University Prognostic Index (CUPI) system to predict survival outcome in hepatocellular carcinoma patients [32, 33]. Previous studies also suggested that coinfection of *S. japonicum* and HBV could lead to accelerated deterioration of hepatic function and higher mortality risk [34, 35]. Abnormal serum AST also reflects the deterioration of hepatic function and could be taken as a surrogate marker for cirrhosis because of reduced plasma clearance of AST secondary to impaired function of sinusoidal cells [36]. Previous studies have also suggested that abnormal DBil was possibly independently associated with an increased risk of hepatic fibrosis, which represented the most common event relevant with decompensating outcome which could lead to death [37, 38]. However, the abnormal DBil is not significantly associated with ASS in our study which could be explained by its lower specificity.

It is an innovation for applying the competing risk analysis method in patients with advanced schistosomiasis after discharge. Competing risk analysis evaluates the informative nature of censoring and the occurrence rates of a particular event, which is more suitable for prognostic analysis. Misleading conclusions might be obtained due to the failure to recognize the presence of competing risks in survival analysis [39]. Due to its rarity of advanced schistosomiasis cases, previous studies focus more on case reports [40, 41]. The strength of our study is that this population-based cohort has a sufficiently large sample size that includes all the local patients with advanced schistosomiasis to build reliable and effective nomograms. The internal and external validation results allow us to generalize the constructed nomograms to a larger population. Moreover, since Chinese government embarked an effort to manage theses cases independently and those registered in the Advanced Schistosomiasis Cases Management System receive an RMB 5000 subsidy yearly per capita for therapy, the patients lost to follow-up was rare [42]. The database, which is supported by government, provides relatively complete patient data including demographic, clinical and follow-up data updated annually, which bring convenience for our research.

Our study also has several limitations. First, weaknesses inherent to the our dataset include lack of some information on some possible prognosis factors that were not routinely collected such as detailed information on treatment variables, life style and four liver fibrosis indicators. It is well-known that different specific surgeries and chemotherapies, unhealthy life styles such as smoking and excessive alcohol consumption influence survival outcomes. Hyaluronic acid (HA), Laminin (LN), Collagen IV (CIV) and Procollagen III (PCIII) may influence the survival outcome as well [43, 44]. The patients’ ultrasonography examination results of liver and spleen were also not complete. However, models without these markers of liver fibrosis and ultrasonography examination results also performed well (all C-indexes >0.70), suggesting that the good performance of our nomograms. Second, the non-advanced schistosomiasis event, as the competing event, still contains a lot of competing events. In this study, we just regarded them as a whole, which might overstate the impact of competing event. But separating them into minute events will make it difficult for analysis because of the small sample size for each event. Third, the study cohort data, which are obtained from the Hubei Province, may not necessarily reflect the prognosis of patients in other districts very well. Although this model’s performance was internally validated with bootstrap approach and externally validated using a retrospective cohort from Huangshi city, it still needs further study using prospective cohorts.

## Conclusion

In conclusion, this is the first effort to present CIFs for 2-year advanced schistosomiasis specific mortality and competing mortality for patients with advanced schistosomiasis after discharge. The effective predictors of 2-year OS and ASS were discovered through competing risk analysis. We further built the nomograms to estimate 2-year OS and ASS. This individualized prognostic predictive tool will help to guide follow-up and aid accurate prognostic assessment.

## Data Availability

Not applicable.

## Abbreviations

OS: Overall survival
ASS: Advanced schistosomiasis-specific survival
TBil: Total bilirubin
DBil: Direct bilirubin
ALT: Alanine aminotransferase
AST: Aspartate aminotransferase
ALP: Alkaline phosphatase
ALB: Albumin
HBsAg: Hepatitis B surface Antigen
AFP: Alpha fetoprotein
NASD: Non-advanced schistosomiasis-specific death
ASD: Advanced schistosomiasis-specific death
CIF: Cumulative incidence function
ASM: Advanced schistosomiasis specific mortality
CM: Competing mortality
